# The connectomes of males and females with autism spectrum disorder have significantly different white matter connectivity densities

**DOI:** 10.1038/srep46401

**Published:** 2017-04-11

**Authors:** Andrei Irimia, Carinna M. Torgerson, Zachary J. Jacokes, John D. Van Horn

**Affiliations:** 1 Laboratory of Neuro Imaging, USC Mark & Mary Stevens Neuroimaging and Informatics Institute, Keck School of Medicine, University of Southern California, 2025 Zonal Avenue, Los Angeles CA 90032 USA

## Abstract

Autism spectrum disorder (ASD) encompasses a set of neurodevelopmental conditions whose striking sex-related disparity (with an estimated male-to-female ratio of 4:1) remains unknown. Here we use magnetic resonance imaging (MRI) and diffusion weighted imaging (DWI) to identify the brain structure correlates of the sex-by-ASD diagnosis interaction in a carefully selected cohort of 110 ASD patients (55 females) and 83 typically-developing (TD) subjects (40 females). The interaction was found to be predicated primarily upon white matter connectivity density innervating, bilaterally, the lateral aspect of the temporal lobe, the temporo-parieto-occipital junction and the medial parietal lobe. By contrast, regional gray matter (GM) thickness and volume are not found to modulate this interaction significantly. When interpreted in the context of previous studies, our findings add considerable weight to three long-standing hypotheses according to which the sex disparity of ASD incidence is (A) due to WM connectivity rather than to GM differences, (B) modulated to a large extent by temporoparietal connectivity, and (C) accompanied by brain function differences driven by these effects. Our results contribute substantially to the task of unraveling the biological mechanisms giving rise to the sex disparity in ASD incidence, whose clinical implications are significant.

One in ~68 children in the US suffers from autism spectrum disorder (ASD), which is a highly-heritable neurodevelopmental mental condition characterized by difficulty in communicating, forming relationships with other individuals, using language and manipulating abstract concepts. The precise nature and spatial profile of structural brain differences between ASD patients and typically developing (TD) subjects has been a topic of debate for at least two decades^1^,^2^. Though the frontal, temporal, occipital lobes and the limbic system develop abnormally in children with ASD, the precise extent of such abnormalities remains insufficiently resolved^1^. It has been proposed^2^ that the behavioral phenotype of ASD is unlikely to be explained by abnormalities which reside in a single brain region, and further suggested that the abnormal brain development associated with ASD may instead involve widely-distributed, non-overlapping areas which are recruited during social information processing tasks. These areas are typically thought to include prefrontal cortex, medial and ventral temporal lobe, the superior temporal sulcus, amygdala and cerebellum, and it is precisely these regions which are also candidate brain determinants of autism^3^,^4^,^5^.

There is a strong sex-related disparity in ASD incidence, with an estimated male-to-female ratio of 4:1 (see ref. 6). The causes of this striking difference remain difficult to explain, partly because of the limited number of neurobiological studies on sex differences in affected individuals. However, this bias may partly also be due to nosologic, diagnostic and to other factors whose effects are particularly challenging to quantify^7^. For example, the extreme male brain (EMB) theory of autism postulates that the perceived sex disparity in ASD incidence is due to sex differences in the domains of empathy (the ability to identify and respond to other individuals’ mental states, an ability of which females allegedly have higher levels) and systemizing (the ability to analyze and construct systems, at which males are ostensibly better)^8^,^9^,^10^. In one study, Nordahl *et al*.^11^ found sex differences in the neuroanatomy of the corpus callosum in longitudinally-studied preschool-aged children with ASD. In another, Beacher *et al*.^10^ investigated the validity of the EMB theory in adults with Asperger’s syndrome (AS), which could be considered a ‘pure’ ASD in that AS does not involve a learning disability or major delay in spoken language development. The latter authors proposed that autism attenuates sex differences in brain structure and provided initial evidence of atypical sexual dimorphism in the brain structure of adults with AS, suggesting that sex-related differences in neurotypical control subjects are attenuated or absent in AS participants. However, individuals with AS were not found to have masculinized brains, which was interpreted as evidence against the core hypothesis of EMB theory. By contrast, Di & Biswal^12^ found solely sex-independent, large-scale neuroanatomical alterations in children with ASD, which were deemed to be consistent with the predictions of EMB theory. On the other hand, Alaerts *et al*.^13^ identified highly-consistent patterns of functional hypo-connectivity in ASD males compared to TD males, and hyper-connectivity in females with ASD compared to TD females. The hyper-connectivity patterns in ASD females were interpreted as reflective of neural masculinization, whereas hypo-connectivity patterns observed in ASD males were found to exhibit the features of neural feminization, which prompted Alaerts *et al*. to propose that, contrary to the predictions of EMB theory, ASD is a disorder of sexual differentiation rather than masculinization in both genders. Such potentially contradictory findings across studies suggest that the ongoing debate regarding the relationship between ASD modulators, ASD diagnosis and brain structure remains far from settled.

Though, as described above, some insight into the structural correlates of the sex-related disparity in ASD incidence has been provided by both structural and functional neuroimaging studies, methodological concerns have been raised about some of them. Factors which have been invoked as reasons for this skepticism include the facts that (A) the neuroimaging literature on sex-related brain structure differences between ASD patients and TD volunteers is reportedly quite limited^14^,^15^,^16^,^17^, and that (B) there is substantial heterogeneity across the ASD phenotype, which may have contributed to the reported failure of some studies to replicate one another’s findings^17^. This suggests that neuroimaging studies of brain structure in ASD should include robust sample sizes to ensure adequate estimates of statistical power, effect sizes and statistical significance. Here, we use magnetic resonance imaging (MRI) and diffusion weighted imaging (DWI) to investigate the effects of sex, ASD diagnosis and their interaction upon regional cortical area, cortical thickness, cortical curvature, gray matter (GM) volume and white matter (WM) connectivity density (CD) in a carefully-selected cohort consisting of 110 ASD and 83 TD volunteers (95 females). We investigate the effects of sex, ASD diagnosis and of their interaction upon the regional values of these neuroimaging metrics using a 2 × 2 factorial design and canonical correlation analysis (CCA) in a multivariate setting. The hypothesis of the study is that there are structural brain features which are significantly different, in a statistical sense, between the study group (ASD) and the control group (TD), on the one hand, and between males and females, on the other hand. The WM connectivity density of temporal and parietal brain areas is found to be modulated, to a statistically significant extent, by the statistical interaction between sex and ASD diagnosis, which suggests that differences in brain circuitry in these brain regions are strongly associated with the sex disparity in ASD incidence. Thus, our results suggest powerful strategies for future research into this puzzling, mysterious and clinically significant phenomenon.

## Results

The information in Table 1 summarizes the results of a demographic analysis applied to our sample. Upon assessing the cognitive abilities of the participants using the Differential Ability Scales (DAS-II), no statistically significant difference in either age or DAS scores was found between the study group (ASD) and the control group (TD). The sex composition between the two samples was not found to differ significantly either (χ^2^ = 0.1304, d. f. = 1, *p* < 0.72). The total intracranial volumes (TICVs) of the two groups were not found to be statistically different (*t* = 1.1437, d. f. = 187.54, *p* < 0.1271).

Subsequent to the acquisition of neuroimaging data from each volunteer, each *T*_*1*_-weighted MRI volume was segmented and a three-dimensional (3D) model of the cortical surface was created to produce an anatomically-faithful, smooth representation of the GM/WM interface (see Methods). For each brain hemisphere, every gyrus and sulcus was delineated, parceled and the local GM thickness, volume, cortical area, mean curvature and CD were calculated. A statistical analysis in four steps was subsequently carried out (see **Methods** for a detailed description). All steps of the analysis are described below and summarized in Table 2.

Step 1 of the statistical analysis involved testing the omnibus statistical hypothesis according to which neither ASD diagnosis, sex nor their interaction had any statistically significant effect upon any of the brain structure properties available to us (i.e. GM thickness, GM volume, cortical area, mean curvature and CD). This hypothesis was not rejected for cortical area (*F*_495,76_ = 1.153, *p* < 0.1673), cortical thickness (*F*_495,76_ = 1.091, *p* < 0.281), cortical curvature (*F*_495,76_ = 1.1436, *p* < 0.183) or for GM volume (*F*_495,76_ = 0.824, *p* < 0.880). The null hypothesis was, however, rejected for CD (*F*_495,76_ = 1.921, *p* < 0.031), suggesting that the statistical main effect observed in our sample was driven by brain features associated with WM connectivity, rather than GM structure.

Step 2 investigated whether the observed main effect on connectivity was due either to diagnosis, sex or to the interaction between the two. The hypotheses according to which the observed connectomic differences between groups were due neither to sex (*F*_3,24_ = 2.162, *p* < 0.119) nor to diagnosis (*F*_3,24_ = 1.662, *p* < 0.202) could not be rejected, whereas the hypothesis that these differences were not due to their interaction was, in fact, rejected (*F*_3,24_ = 3.675, *p* < 0.026). This indicates that sex-related disparities between ASD and TD volunteers are associated, in a correlative sense, with CD differences between these groups.

In Step 3, we used CCA to confirm that no statistically-significant correlation existed between CD and either diagnosis (*F*_165,52_ = 1.097, *p* < 0.356) or sex (*F*_165,52_ = 1.175, *p* < 0.253), but that a significant correlation did exist between CD and the sex-by-diagnosis interaction (*F*_165,52_ = 1.794, *p* < 0.008). The analysis in Step 3 confirms the main finding in Step 2, according to which the observed connectomic differences between groups are due to sex-by-diagnosis interactions. Importantly, the fact that this conclusion was reached using two distinct inferential approaches (multivariate regression in Step 2 vs. CCA in Step 3) lends additional credence to the soundness of our statistical analysis.

**Figure 1:**
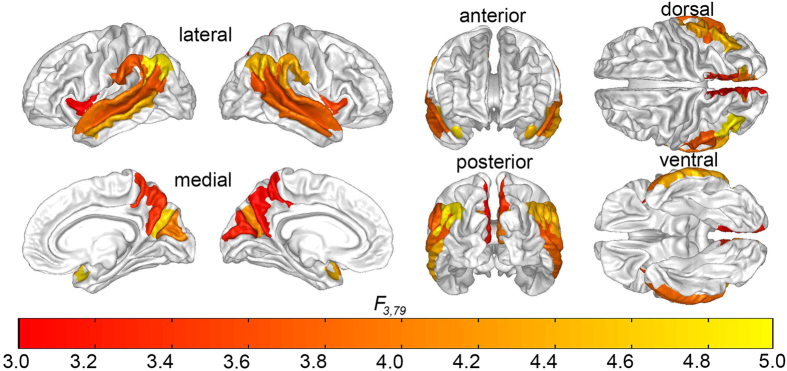
Contributions of each brain region to the statistical significance of the sex-by-diagnosis interaction on CD inferred using LOO CCA (see text). Partial *F* statistics with 3 and 79 d. f. are color-coded on the cortical surface of an average brain, at locations associated with each region whose corresponding response variable (brain region) had been removed from the CCA. The blue vertical bar in the horizontal scale indicates a value of *F*_3,79_ = 3.152, corresponding to an FDR value of 0.05. Brain regions drawn in shades of orange and yellow displayed to the right of the vertical bar contribute significantly to the sex-by-diagnosis interaction in the studied cohort.

In Step 4, we tested the null hypothesis that the CD of each *individual* cortical parcel did not contribute significantly to the main effect of the sex-by-diagnosis interaction upon CD. This step of the analysis allowed us to identify the relative contribution of each brain region to the main effect which had been found in Step 2 and confirmed in Step 3. In Step 4, the partial-*F* statistic associated with the statistical test involving each cortical parcel was color-coded and displayed on the cortical surface of an average brain at the corresponding location of the respective cortical region whose contribution to the main effect had been tested. The results of this analysis are shown in Fig. 1, which indicates that the brain regions which are responsible to the greatest extent for sex-by-diagnosis interaction effects on CD are located bilaterally on the lateral aspect of the temporal lobe (superior temporal gyrus, middle temporal gyrus, superior temporal sulcus), at the temporo-parieto-occipital junction (subcentral gyrus, supramarginal gyrus) and in the medial parietal lobe (precuneus, parieto-occipital sulcus).

## Discussion

Sex-related differences in the brain structure of ASD subjects have not been investigated closely enough to determine how such differences modulate the sex-related disparity of ASD. Specifically, how the structural differences identified here might modulate brain function and thereby affect ASD sex disparity is of substantial interest. In this study, the lateral aspect of the temporal lobe, the medial aspect of the parietal lobe and the temporo-parieto-occipital junction were all identified as regions which modulate the statistical interaction between sex and ASD diagnosis. In what follows, we discuss the potential implications of our findings as they relate to each of these three structures.

The lateral aspect of the temporal lobe—particularly the superior temporal gyrus (STG, i.e. Wernicke’s area, Brodmann’s area 22p) and its posterior portion (Brodmann areas 41 and 42)—have key roles in processing a wide variety of auditory stimuli (including speech) as well as in social cognition. In agreement with our findings, an influential study by Bigler *et al*.^18^ found that ASD and TD subjects exhibited differences in STG volume and that these differences persisted even when education, IQ and head size were taken into account. Furthermore, when examining the relationship between STG volume differences and intellectual ability, Bigler *et al*. concluded that STG-related structural differences between ASD and TD subjects were also associated with differences in language development trajectories between these two groups. Based on this evidence, the authors concluded that autism may involve a possible failure in left hemisphere lateralization of language function involving the STG. When interpreted in this context, our own findings suggest that the potential developmental failure proposed by Bigler *et al*. may preferentially target males rather than females, and that this phenomenon could be one of the possible causes of the sex disparity observed in ASD. To test these important hypotheses, future studies should aim to identify and describe how sex modulates the expression of genes which control STG maturation during development.

The medial parietal lobe is known to play a prominent role in self-awareness^19^, consciousness^20^ and memory processing^21^. In particular, the central region of the precuneus is heavily connected to the temporo-parieto-occipital junction (also of interest here) by white matter tracts whose architecture is shared across primates^22^. This brain region appears to be heavily involved in both executive function and motor planning^23^, and differences in its structure between ASD and TD subjects have been documented both extensively and reliably according to meta-analytic studies^24^. Compared to TC volunteers, ASD subjects have substantial alterations in functional connectivity between the medial parietal lobe and many other brain regions during tasks which recruit their ability to perceive and interpret social interactions^25^. These and similar findings may suggest that the sex disparity of ASD is partly associated with widely-distributed fractionation of social brain circuits, which has also been studied extensively^26^.

The angular gyrus is located close to the junction of the temporal, parietal and occipital lobes and, as a member of both the rich club^27^ and core scaffold^28^ of the human connectome, this structure serves a very prominent role in both structural and functional brain networks. As a prominent connectivity hub, this gyrus and some of its adjacent structures—particularly supramarginal cortex—play an essential role in a variety of complex, high-level tasks which recruit numerous areas of the brain. For example, multisensory integration^29^,^30^, comprehension of metaphoric or other symbolic speech^31^, analytic and spatial cognition^32^, spatiovisual attention^29^,^30^ and awareness^33^ all recruit the angular gyrus. Given that connectopathies appear to modulate autistic behavior substantially^26^, the indication that the angular gyrus modulates the interaction of sex and ASD diagnosis is perhaps unsurprising.

Interestingly, default mode network (DMN) activity in parietal and ventromedial temporal structures exhibits significant sex-related differences in ASD subjects^34^. When interpreted in this context, our findings appear to add weight to the long-standing theory that parietal and temporal cortices plays a prominent role in modulating sex-related differences in the brain structure of ASD subjects^35^,^36^. Our results also lend credence to the hypothesis that brain circuits which differ in ASD and TD subjects due to sex-by-diagnosis interaction effects also exhibit functional differences driven by this type of effects. If this is true, our work could greatly help to unravel the biological mechanisms which give rise to the observed disparity of ASD incidence between sexes.

Among previous studies, that of Schaer *et al*.^17^ appears to be most similar to ours because it (A) features a large enough sample size to achieve acceptable statistical power, (B) partials out statistical effects due to intelligence quotient (IQ) and head size, and (C) explores sex-by-diagnosis interactions explicitly based on structural measures obtained using a neuroimaging analysis workflow similar to ours. As in our study, Schaer *et al*. did not identify a significant main effect of sex-by-diagnosis interaction when comparing the local cortical volumes and cortical thickness values of ASD vs. TD subjects.

In the case of local gyrification (a measure strongly dependent upon local curvature), Schaer *et al*. did find a sex-by-diagnosis interaction in a small portion of right orbitofrontal cortex, though no main effect predicated on either sex or diagnosis was identified. Interestingly, Lai *et al*.^16^ also failed to find a significant sex-by-diagnosis interaction on GM volume, though they did identify a sex-by-diagnosis interaction on WM volume in the medial parietal lobe and along the inferior longitudinal fasciculus of both hemispheres. Similarly, in our study, the WM CDs of the medial parietal lobe and of the lateral temporal lobe were found to exhibit sex-by-interaction effects, indicating agreement between our study and that of Lai *et al*. Because cortical gyrification differs significantly between ASD and TD males^37^, our inferences and those of Schaer *et al*. suggest that males and females with ASD share similarly abnormal gyrification.

In 2016, Krishnan *et al*.^38^ studied ASD-associated genetic changes in the spatiotemporal development of the brain as well as the enrichment of prominent gene expression signatures (sets of genes highly expressed in a certain brain region) in a genome-wide ranking of ASD genes. For gene expression signatures localized in the temporal and medial parietal lobes, this study found a surprising prenatal signal which indicates, according to the authors, the presence of a major effect of ASD-associated mutations upon the fetal development of the temporal and parietal lobes. In the context of this novel finding, our own results suggest that a relationship may exist between the perceived sex-related disparity in ASD incidence, on the one hand, and the effect of genetic mutations upon the temporal and parietal lobes during pre-natal development, on the other hand.

The task of interpreting and comparing imaging studies of brain structure differences between ASD and TD subjects is challenging in several important ways. On the one hand, a substantial number of studies attempting to characterize specific ASD-related abnormalities in the structure of frontal, temporal and parietal cortices have not been replicated, possibly due to small population sample sizes analyzed in some previous neuroimaging studies of ASD^39^,^40^. On the other hand, differences in data acquisition methods, image processing, diagnosis/inclusion criteria, age, IQ and physical health have all been proposed as causes of contradictory findings across neuroimaging studies of ASD^2^. The potential presence of such confounds is particularly worrisome due to the highly heterogeneous nature of ASD, where genes, structural brain phenotype and behavior interact throughout development^41^. In light of the above, four important advantages of the present study are that (A) it does provide confirmation of findings in two important previous studies^17^,^34^, as discussed above, (B) it uses a carefully-selected sample of matched ASD and TD subjects whose neuroimaging data were acquired using an identical MRI protocol at all participating sites, (C) it features a balanced statistical design and (D) it includes a larger sample of females with ASD than in many previous study. Future studies should also strive to use adequate sample sizes which can afford a solid foundation for any statistical inferences drawn from their data. Potentially larger head motion in ASD subjects compared to TD volunteers^42^, though unaccounted for here, should also be taken into account by future studies.

In conclusion, our findings are in agreement with previous studies which indicate that no sex-by-diagnosis interaction effects on volumetric measures exist in ASD subjects, whereas interaction effects on WM connectivity are in fact present in parietal and temporal regions. When considered in the context of functional connectivity differences between males and females with ASD, our results suggest that future research should focus on applying connectomic approaches to the systematic mapping and study of both structural and functional brain circuitries in parietal and temporal regions. For example, one particularly interesting hypothesis which should be explored is whether perceived differences in ASD incidence among males vs. females are due to altered structural/functional connectivity in temporo-parietal regions, and whether such alterations are either the consequence or the result of differences in cognition, affect and behavior between male and female ASD patients.

## Methods

### Participants

The study was undertaken as part of the Autism Center of Excellence (ACE) Program, which is funded by the National Institute of Mental Health (NIMH). Neuroimaging data were acquired at one of four sites: (1) the Center for Translational Developmental Neuroscience, Child Study Center, Yale School of Medicine, New Haven, CT (73 subjects); (2) the Nelson Laboratory of Cognitive Neuroscience, Boston Children’s Hospital, Harvard Medical School, Boston, MA (49 subjects); (3) the Center on Human Development & Disability, Seattle Children’s Hospital, University of Washington School of Medicine, Seattle, WA (92 subjects); (4) Staglin IMHRO Center for Cognitive Neuroscience, David Geffen School of Medicine, University of California, Los Angeles, CA (73 subjects). The research study was undertaken in agreement with US federal law (45 CFR 46) and was approved by the Institutional Review Boards (IRBs) at each of the four institutions where human volunteer data were acquired. All methods were implemented in accordance with the relevant guidelines and regulations of the IRB at the Keck School of Medicine of USC, which approved this study. Volunteers were recruited while aiming for an adequately balanced statistical study design, including *N*_*1*_ = 110 ASD patients (55 females) and *N*_*2*_ = 83 TD subjects (40 females), for a total of *N* = *N*_*1*_ + *N*_*2*_ = 193 volunteers. Informed consent was obtained from all subjects and from their legally authorized representatives. The cognitive abilities of the participants (i.e. their IQ) were assessed using the Differential Ability Scales (DAS-II), including the Verbal (V), Non-Verbal (NV), Spatial (S), General Conceptual Ability (GCA) and Spatial Non-Verbal Composite (SNC) scales. The significance of differences in age and DAS scores between the two cohorts was evaluated using Welch’s *t* test for samples with unequal variances. Sex was coded as a binary variable, using +1 for males and −1 for females, such that a cohort with an equal number of males and females would have a mean value of 0 for the sex-coding variable. The statistical significance of the difference in sex composition between the two groups was evaluated using a χ^2^ test.

### Inclusion criteria

Inclusion of ASD patients was made based on the Autism Diagnostic Interview (ADI) and on the Autism Diagnostic Observation Schedule (ADOS-2). For the ADI, inclusion criteria were: (*i*) a communication total (R) score greater than 8; (*ii*) a behavioral (S) total score greater than 6; (*iii*) a social affect (T) total score greater than 1; (*iv*) a sum of the previous three greater than 18, i.e. R + S + T > 18. For the ADOS, the inclusion criterion was a comparison score greater than 3. Both ADI and ADOS criteria had to be satisfied for inclusion.

### Exclusion criteria

For the ASD group, these included the presence of any genetic, neurological or psychiatric comorbidity, including but not limited to fragile X syndrome, epilepsy, spasms, brain damage, pre-/peri-natal birth injury, severe nutritional or psychological deprivation, visual or auditory impairment after correction, sensorimotor difficulties precluding valid use of diagnostic instruments, use of any benzodiazepine, barbiturate or anti-epileptic medication, pregnancy and active tic disorders. Exclusion criteria for the TD group also included diagnosed, referred or suspected ASD, schizophrenia, learning/intellectual disability, any other developmental or psychiatric disorders as well as having a first- or second-degree relative with ASD.

### Recruitment protocol

Potential enrollees were screened by research-reliable clinicians either by telephone or in person to ensure that inclusion/exclusion criteria were satisfied. Phone interviews with parents were conducted prior to enrollee visits. ADI and ADOS measures were collected via direct observation during in-person visits by examiners with research-levels of ADOS and/or ADI reliability, as appropriate. Medical history information was collected during interviews.

### MRI acquisition

MRI volumes were acquired using the same scanner type (Siemens Magnetom TrioTim), magnetic field strength (3 T) and acquisition protocol across all sites. *T*_*1*_-weighted volumes were acquired using a magnetization-prepared rapid acquisition gradient echo (MPRAGE) sequence with the following parameters: 256 sagittally-oriented, single-shot, interleaved slices; 256 × 256 acquisition matrix; 256 mm field of view (FOV); 1 mm slice thickness; 2530 ms repetition time (*T*_*R*_); 3.31 ms echo time (*T*_*E*_); 1100 ms inversion time (*T*_*I*_); 7-degree flip angle; 100% phase and slice resolutions; 200 Hz/pixel (Px) bandwidth and 7.6 ms echo spacing. DWI volumes were acquired in 64 diffusion gradient directions using the following acquisition parameters: 60 transversally-oriented, interleaved slices; 96 × 96 acquisition matrix; 190 mm FOV; 2 mm slice thickness; 9000 ms TR; 93 ms TE; 7-degree flip angle; 100% phase resolution; *B*_*0*_ values of 0 and 1000 s/mm^2^; 2264 Hz/Px bandwidth and 0.69 ms echo spacing. It should be noted that the ACE protocol for DWI data acquisition is not a high angular resolution diffusion imaging (HARDI) protocol. All neuroimaging data were de-identified, encrypted and then transferred to the Data Coordinating Center (DCC), which resides at the Laboratory of Neuro Imaging (LONI) in the Mark & Mary Stevens Neuroimaging and Informatics Institute at University of Southern California (USC). Protocol compliance and quality control (QC) were undertaken using the LONI QC System (http://qc.loni.usc.edu). Data were also stored in the National Database for Autism Research (NDAR, http://ndar.nih.gov).

### Image processing

All image processing was undertaken using the LONI Pipeline environment (http://pipeline.loni.usc.edu). First, for each subject, DWI and MRI volumes were affinely co-registered. Eddy current correction was then applied to each DWI volume, which was subsequently processed in TrackVis 6.0 (http://trackvis.org) using its Diffusion Toolkit 0.6 utility to correct for the oblique scan prescription of the Siemens MRI scanner. Tensors were then fit to the DWI data to perform diffusion tensor imaging using default parameters and to then reconstruct fiber streamlines using deterministic tractography. The algorithm used for the latter was fixed step-length streamline propagation^43^. Individual streamlines exhibiting a turning angle below 60 degrees were discarded. The cortical surface of the brain was reconstructed as a triangular tessellation with ~300,000 vertices (average inter-vertex distance: ~1 mm) to produce an anatomically faithful, smooth representation of the GM/WM interface^44^. At each vertex *v*_*i*_ of the tessellation, cortical thickness was measured in FreeSurfer 6.0 as the distance between the GM/WM boundary and the brain surface. For each brain hemisphere, a total of 74 cortical structures (gyri and sulci) were identified and parceled in FreeSurfer 6.0 using a probabilistic atlas, as described elsewhere^45^. The brain stem was also segmented in FreeSurfer as well, for a total of 165 parcels over the entire brain. Neuroanatomical labels were assigned to voxels based on probabilistic information estimated from a manually labeled training set. The method uses the previous probability of a tissue class occurring at a specific atlas location and the probability of the local spatial configuration of labels given each tissue class. The technique is comparable in accuracy with manual labeling^46^. Subsequent to parcellation, the surface area, volume and mean curvature of each structure were calculated. The white matter connectivity calculation was performed as described elsewhere^47^,^48^,^49^.

### Statistical analysis

Prior to statistical analysis, every measure was normalized by the total intracranial volume of the corresponding participant in question in order to avoid the confounding effect of head size. Then, the statistical effects due to age, acquisition site, intelligence (as quantified using the DAS) and to the interaction between the three were partialed out from the linear regression and all subsequent analyses were based on the residuals from this regression. To investigate the potential statistical significance of age-related nonlinear effects upon brain structure (which have been reported elsewhere ref. 50), the design matrix initially included the square of each volunteer’s age and the null hypothesis that the regression coefficient associated with the square of age was equal to zero was tested in the context of a LOO (reduced model) analysis as described elsewhere (see pp. 330–331 in Rencher ref. 51). After correction for multiple comparisons, this hypothesis could not be rejected at the *α* = 0.05 significance level (see **Results**), which prompted us to discard the square of age as an independent variable and to continue our analysis without this term in the design matrix. Instead, only age was included in the model. We sought to identify those structural brain features which were significantly different, in a statistical sense, between the study group (ASD) and the control group (TD), on the one hand, and between males and females, on the other hand. For each subject in the study, the image processing steps detailed above provided values for five neuroimaging metrics (GM thickness, volume, cortical area, mean curvature and CD) in the brain regions parceled. A fixed-effects, multivariate multiple correlation analysis was then implemented as follows. First, a fixed-effects design matrix ***X*** was generated which contained sex, diagnosis (ASD or TD) and their interaction as predictor variables. The number of predictor variables is denoted here by *q*, hence *q* = 3 in this study. Secondly, for each of five neuroimaging metrics listed above, a distinct response matrix ***Y***_*i*_ (*i* *=* 1, …, 5) was created which contained the values of one of the 5 neuroimaging metrics (e.g. GM thickness) for each of the *p* brain regions. It should be noted that ***X*** is identical (fixed) for all *i*. The statistical analysis approach outlined below closely follows that of Rencher^51^, to whose treatise the reader is referred for details.

The statistical analysis was implemented in four distinct steps. In Step 1, the null hypothesis that diagnosis, sex or their interaction in ***X*** had no statistically significant omnibus effect upon the response variables in each matrix ***Y***_*i*_ was tested. The matrix ***B***_*i*_, containing the least-squares estimators 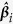 to the set of multivariate regression equations for neuroimaging metric *i*, is given by


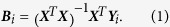


Furthermore, for each neuroimaging metric *i*, the hypothesis and error matrices ***H***_*i*_ and ***E***_*i*_ are, respectively,


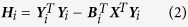


and


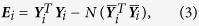


where (⨪) denotes the mean over subjects. Let *v*_*H*_ = *q* be the degrees of freedom (d. f.) for ***H*** and *v*_*E*_ = *N* − *q* − 1 be the d. f. for ***E***, and *p* be the number of response variables. With


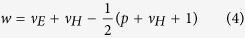


and


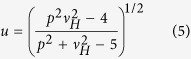


the omnibus test statistic is Wilks’ *Λ*, defined as 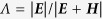, which can be converted to an *F* statistic with *d*_1_ and *d*_2_ d. f. via the transformation


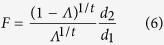


The degrees of freedom *d*_1_ and *d*_2_ are given by


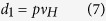



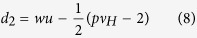


Null hypotheses were rejected at a significance level α equal to 0.05.

Steps 2–4 of the statistical analysis were only implemented whenever the null hypothesis could not be accepted in Step 1. In Step 2, a LOO (i.e. reduced model), fixed effects, multivariate, multiple regression analysis was performed. Specifically, for each of the *q* = 3 predictor variables in ***X***, a reduced-model design matrix ***X***_*j*_ (*j* = 1, …, *q*) was created by deleting exactly one of the predictor variables (sex, diagnosis or their interaction) from both of ***X*** and ***B***_*i*_. In this LOO model, the partial *Λ* statistic can be converted to a partial *F* statistic using the exact transformation


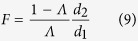


where *F* has d. f. equal to *d*_1_ = *v*_*H*_ and *d*_2_ = *v*_*E*_ − *p*.

In the case of any neuroimaging variable for which the omnibus null hypothesis could not be accepted, Step 3 of the statistical analysis aimed to identify which predictor variable (diagnosis, sex or their interaction) contributed most substantially to the canonical correlation between predictor variables and each of the response variables being considered. To infer this, a canonical correlation analysis (CCA) was implemented following the approach outlined by Rencher^51^, as summarized below. For convenience, the subscripts *i* and *j* for ***Y***_*i*_ and ***X***_*j*_, respectively, are henceforward omitted for convenience and should be assumed to be implicit. Briefly, the overall sample covariance matrix ***S*** for ***Y*** and ***X*** can be partitioned as


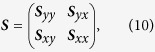


where ***S***_*yy*_ is the *p* × *p* sample covariance matrix of ***Y**, **S***_*yx*_ is the *p* × *q* sample covariance between ***Y*** and ***X***, and ***S***_*xx*_ is the *q* × *q* sample covariance matrix of ***X***. With *s* = min(*p, q*), the square roots *r*_*k*_ (*k* = 1, …, *s*) of the eigenvalues associated with the matrix 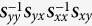 are the canonical correlations (measures of association) being sought. The quantity 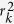 is the maximum squared correlation between a linear combination *u*_*k*_ = **a**^*T*^***y*** and another linear combination *v*_*k*_ = **b**^*T*^***x***, which are called the *canonical variates* of the data set. The eigenvalues of 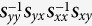 can be obtained from the characteristic equations


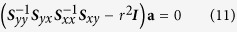



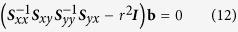


where ***I*** is the identity matrix. Firstly, simultaneous diagonalization^52^,^53^ was used to obtain the eigenvalues and eigenvectors of these equations. Subsequently, a LOO analysis was implemented to explore the effect of removing each predictor variable from the system of canonical equations. For this LOO analysis, *v*_*H*_ = *q* − 1 and the partial *F* statistic can be computed as in step 2.

It should be noted that the statistical inferences made in Steps 2 and 3 are equivalent despite the fact that two distinct statistical inference approaches were used (multivariate regression in Step 2 vs. CCA in Step 3). One reason for implementing Step 3 is that the CCA sets the stage for and facilitates the process of testing the statistical hypotheses in Step 4, whereas the multivariate regression model in Steps 1–2 is not ideal for this purpose.

Step 4 involved carrying out a LOO analysis to investigate the effect of removing each response variable (as opposed to predictor variable, as in Step 3) from the system of canonical equations. The details of the analysis are as described in Step 2, though using *p*’ = *p* − 1 to account for the removal of a single response variable from the model. To quantify the effect of removing each response variable from the system of canonical equations, each partial *Λ* statistic was converted to a partial *F* statistic and the null hypothesis that the removal of the response variable in question had no significant effect upon the canonical correlation was tested. The test statistic is a partial Wilks’ *Λ*, which was converted to a partial *F* statistic as previously explained. For all steps of the statistical analysis in this study, corrections for multiple comparisons were implemented using the false discovery rate (FDR) approach of Benjamini & Hochberg^54^ because no statistical test was found to survive the Bonferroni correction. The statistical analysis was implemented in MATLAB (www.mathworks.com) following the approach of Rencher^51^. As pointed out by an anonymous reviewer, controlling for multiple comparisons using the FDR approach does not also control for the family-wise error rate (FWER), which would be useful in this study. For this reason, an alternative approach such as the implementation of maximum cluster permutation statistics may have been a preferable alternative. Consequently, the use of the FDR approach should be acknowledged as a limitation of the statistical analysis in this study.

Our adoption of a multivariate approach to the statistical analysis implemented in this study is motivated by the fact that, in each step of the analysis, the goal was to test a relatively large number of null hypotheses pertaining to structural brain differences between the volunteer groups studied. For this reason, a multivariate approach is preferable to a univariate one for the following important reasons, all of which are discussed in detail by Rencher^51^: (A) the use of univariate tests inflates the Type I error rate *α*, whereas multivariate tests preserve the exact *α* level; (B) univariate tests ignore the correlations among variables, whereas multivariate tests use these correlations directly; (C) multivariate tests often have higher statistical power, and (D) the multivariate tests used here involve the construction of linear combinations of variables which provide more insight than univariate tests as to how the variables themselves unite to test the hypothesis in question. Had we preferred to gain insight into how the sex-by-diagnosis interaction is related to the structure of only a relatively small subset of brain regions, univariate tests may have been preferable.

## Additional Information

**How to cite this article**: Irimia, A. *et al*. The connectomes of males and females with autism spectrum disorder have significantly different white matter connectivity densities. *Sci. Rep.* **7**, 46401; doi: 10.1038/srep46401 (2017).

**Publisher's note:** Springer Nature remains neutral with regard to jurisdictional claims in published maps and institutional affiliations.

## Figures and Tables

**Table 1:** Demographics of the ASD and TD cohorts.

descriptor	ASD			TD			statistics	
	μ	σ	range	μ	σ	range	*t*	*p*
age [yrs]	12.32	3.46	7–18	12.76	3.57	8–18	−0.86	0.39
TICV [cm^3^]	212.94	30.07	151–347	217.56	25.94	151–305	1.14	0.13
DAS-V	103.12	30.36	52–155	98.61	27.69	74–159	1.07	0.28
DAS-NV	100.83	29.46	57–149	97.77	25.79	74–147	0.72	0.48
DAS-S	98.34	28.44	58–154	98.65	24.97	68–144	−0.44	0.66
DAS-GCA	101.94	29.88	62–162	98.16	26.85	79–147	0.92	0.36
DAS-SRC	99.57	30.04	59–158	97.47	25.97	63–139	0.51	0.61

The mean μ and standard deviation σ of each descriptor variable are displayed. Welch’s *t* statistics for samples with unequal variances are reported, as are the corresponding *p* values. For all statistical tests, there are *N* – 2 = 191 d. f. The sex ratio (males to females) for the ASD group is 1:1, whilst for the TD group it is 1.075:1. *Abbreviations*: yrs = years; TICV = total intracranial volume; DAS = differential ability scales; V = verbal; NV = non-verbal; S = spatial; GCA = general conceptual ability; SNC = spatial non-verbal composite. *Symbols*: μ = mean; σ = standard deviation; *t* = *t*-statistic; *p* = *p*-value.

**Table 2:** Statistical analysis summary.

Step 1				
*Test variable*	*F*_495,76_	*p*		
cortical area	1.153	0.167*		
cortical thickness	1.091	0.281*		
cortical curvature	1.144	0.183*		
cortical volume	0.824	0.880*		
CD	1.921	0.031*		
Step 2				
* test variable*	*F*_3,24_	*p*		
sex	2.162	0.119*		
diagnosis	1.662	0.202*		
sex × diagnosis	3.675	0.026*		
Step 3				
* test variable*	*F*_165,52_	*p*		
sex	1.175	0.253*		
diagnosis	1.097	0.356*		
sex × diagnosis	1.794	0.008*		
	***hemisphere***			
**Step 4**	***left***		***right***	
***region associated with the test variable***	**F3,79**	***p***	**F3,79**	***p***
angular gyrus	4.920	0.003	4.369	0.007
parieto-occipital sulcus	4.001	0.010	4.562	0.005
middle temporal gyrus	4.317	0.007	3.797	0.013
supramarginal gyrus	3.680	0.015	4.271	0.008
cuneus	3.185	0.028	3.934	0.011
superior temporal gyrus, lateral aspect	3.892	0.012	3.771	0.014
Jensen’s sulcus	3.843	0.013	3.663	0.016
superior temporal sulcus	3.680	0.015	3.627	0.017
short insular gyrus	2.960	0.037	3.466	0.020
precuneus	3.043	0.034	3.300	0.025
superior temporal gyrus, *planum polare*	3.016	0.035	3.230	0.027

All *p*-values were corrected for multiple comparisons at the *α* < 0.05 level of statistical significance using the Benjamini-Hochberg procedure^54^ (see **Methods** and **Results** for details). For Steps 1–3, *p*-values associated with statistically significant findings are indicated by an asterisk. For Step 4, for brevity, only statistically significant findings are listed in descending order by *F* statistic and the asterisks are omitted.
